# Barriers and facilitators to implementing an integrated electronic health records system to improve tuberculosis preventive treatment among people living with HIV: a content analysis study from Georgia

**DOI:** 10.3389/fdgth.2026.1754076

**Published:** 2026-05-04

**Authors:** Mariana Buziashvili, Akaki Abutidze, Otar Chokoshvili, Zaza Avaliani, Nata Kazakhashvili, Jack DeHovitz, Mamuka Djibuti

**Affiliations:** 1 Scientific Research Unit, National Center for Tuberculosis and Lung Diseases, Tbilisi, Georgia; 2 Department of Medicine, Division of Public Health, Tbilisi State University, Tbilisi, Georgia; 3 T. Tsertsvadze Infectious Diseases, AIDS, and Clinical Immunology Research Center, Tbilisi, Georgia; 4 Department of Medicine, European University, Tbilisi, Georgia; 5 Division of Infectious Diseases, State University of New York Downstate Health Sciences University, Brooklyn, NY, United States; 6 Partnership for Research and Action for Health, Tbilisi, Georgia

**Keywords:** digital health, electronic health records (EHR), HIV, tuberculosis, tuberculosis preventive treatment

## Abstract

**Introduction:**

The integration of tuberculosis (TB) preventive treatment (TPT) into HIV services through electronic health records (EHR) can improve outcomes by enhancing care coordination, reducing redundancies, and supporting data-driven decision-making. In Georgia, despite close collaboration between TB and HIV programs, service delivery and data systems remain siloed, forcing patients to navigate between facilities and limiting the effectiveness of TPT among people living with HIV (PWH).

**Methods:**

We conducted a qualitative study guided by the Consolidated Framework for Implementation Research (CFIR). Semi-structured interviews with 20 healthcare workers of diverse backgrounds were analyzed using deductive content analysis, structured around the five CFIR domains. The study explored the barriers and facilitators to implementing an integrated EHR system for TB and HIV programs in Georgia, with a focus on improving TPT delivery. Findings aim to inform strategies to enhance digital infrastructure and reduce TB burden among PWH.

**Results:**

Participants identified several facilitators, including the perceived advantage of integrated EHR for care continuity among PWH, enhanced patient monitoring, and alignment with international donor priorities. Key barriers included concerns over confidentiality and HIV-related stigma, legal constraints, limited financial and human resources, and insufficient infrastructure such as outdated equipment and unreliable connectivity. Workforce-related challenges, including digital literacy gaps among older clinicians and increased workload during system transition, were also noted. Respondents emphasized the need for phased implementation, stakeholder engagement, and tailored training to support adoption and long-term sustainability.

**Conclusions:**

Strong support exists for the implementation of an integrated EHR to improve TPT among PWH in Georgia, but significant barriers must be addressed, including confidentiality, financing, governance, and training needs. Integrating TPT into HIV services, with an EHR module designed to support patient-centered pathways, could reduce many of these challenges. A phased, context-specific approach, supported by continuous feedback mechanisms, will be critical for sustainable EHR implementation.

## Introduction

The rapid advancement of digital health technologies is reshaping healthcare delivery across the globe, with electronic health records (EHR) emerging as a cornerstone in enhancing the efficiency of patient care, facilitating coordination among providers, and enabling data-informed clinical decision-making (1). EHRs usually serve as structured digital repositories that capture patients' medical histories, treatment plans, and clinical outcomes, allowing seamless health information sharing across multiple providers and facilities (2–4). Beyond streamlining workflows and reducing the likelihood of medical errors, these systems have also proven valuable in resource-limited settings, where EHRs have shown promise in improving continuity of care, effectively integrating services, and supporting long-term management of chronic conditions through enhanced patient tracking (5–9).

The potential of EHRs extends to public health interventions where integrated service delivery and coordination is critical. In the context of human immunodeficiency virus (HIV) and tuberculosis (TB)—two closely linked epidemics—EHRs offer an opportunity to align programmatic goals and improve patient outcomes. TB remains a leading cause of morbidity and mortality among people living with HIV (PWH), often exacerbated by delayed diagnoses and treatment, including preventive treatment, which is frequently worsened by disjointed healthcare and information systems, as well as inadequate documentation of treatment follow-up (8, 9). Despite WHO recommendations to integrate TB prevention and treatment into routine HIV care and relevant information systems, separate structures persist in many low- and middle-income countries, limiting the effectiveness of TB-related efforts among PWH (10–12).

Although TB preventive treatment (TPT) is a critical intervention to reduce TB-related morbidity and mortality in this population (13–16), its implementation is hindered by fragmented data systems, inconsistent reporting, and the absence of centralized digital infrastructure (17–19). In this setting, an integrated EHR system could potentially support and improve TPT initiation and completion by enabling timely data sharing, enhancing patient monitoring, and facilitating streamlined care coordination, while addressing broader gaps in care coordination between TB and HIV services.

In Georgia, the Ministry of Health has introduced a national electronic health record platform requiring providers to document all outpatient, inpatient, and day-care service information for every citizen. However, there is no separate Health Management Information System unit dedicated specifically to TB or HIV programs. Instead, each program has a separate electronic database with a designated database manager responsible for overseeing and maintaining the respective disease-specific database. Despite this fragmentation, the broader eHealth landscape of the country provides a favorable foundation for future integration of TB and HIV records into a unified platform.

Recent national analyses have demonstrated suboptimal TPT initiation and completion rates among PWH in Georgia, with persistent gaps across the cascade of care (20, 21). In the mixed-methods cascade study, fragmented documentation and limited coordination between TB and HIV services emerged as key barriers, and stakeholders identified digital integration as a potential strategy to address these gaps (20). The present study builds on those findings by further examining the feasibility and implementation considerations of electronic system-based integration to address many of these barriers within the TB and HIV care continuum.

The primary aim of the study was to explore the barriers and facilitators to implementing an integrated and interoperable EHR system for TB and HIV programs in Georgia. Using the Consolidated Framework for Implementation Research (CFIR) (22), we systematically assessed the perspectives of key stakeholders to identify potential implementation challenges and opportunities. Importantly, the study did not assess the national EHR system or the potential integration of a TB/HIV EHR into the national platform. The objective was solely to explore stakeholder perspectives in the potential development and implementation of a TB/HIV-focused EHR system. By generating context-specific insights, this research seeks to inform strategies to enhance digital infrastructure, improve TPT delivery, and ultimately reduce TB burden among PWH in Georgia.

## Methods

### Study design

This study used a qualitative descriptive design guided by the Consolidated Framework for Implementation Research (CFIR), which served as a guiding theoretical structure for data collection and analysis. CFIR is a widely applied implementation science framework that identifies multilevel determinants across five domains influencing implementation processes (22, 23). CFIR was selected for its comprehensive structure and relevance for evaluating health system-level interventions. The interview guide was developed by mapping questions directly to CFIR constructs, ensuring systemic exploration of barriers and facilitators across its five domains (see Supplementary Material, Interview Guide).

### Study settings

Georgia is a middle-income country with a population of approximately 3.7 million. TB and HIV services are delivered through specialized national and regional centers, with centralized oversight and designated regional hubs. Although the Ministry of Health introduced a national EHR platform, disease-specific programs such as TB and HIV continue to operate parallel electronic databases with limited interoperability. The current study involved key representatives from major TB and HIV service providers in Georgia, including the T. Tsertsvadze Infectious Diseases, AIDS, and Clinical Immunology Research Center (IDACIRC) in Tbilisi; central HIV hubs in major regional cities of Kutaisi, Batumi, and Zugdidi; the National Center for Tuberculosis and Lung Diseases (NCTLD); and the National Center for Disease Control and Public Health (NCDC&PH). The electronic systems of both programs capture demographic, epidemiological, clinical, and laboratory data, including TPT-specific information. Since TPT is initiated at TB centers, the HIV system's module is rarely used, and data exchange between platforms remains fragmented, relying mostly on informal communication.

### Study population

Participants were recruited using purposive sampling to ensure representation of diverse roles and perspectives, including clinicians, nurses, technical experts, individuals with TB/HIV program oversight and operational responsibilities, and policymakers directly or indirectly involved in TPT and HIV care. Given the small and well-defined professional community engaged in TPT in Georgia, sampling aimed to include the full pool of eligible stakeholders rather than a subset. A total of 20 individuals were approached and interviewed. Sampling aimed to capture variation across geographic regions, levels of clinical responsibility, and programmatic involvement. Interviews concluded once the available pool of credible and relevant stakeholders had been fully represented and additional participants were unlikely to contribute new or distinct insights. To preserve confidentiality within this small professional community, respondent roles are reported using aggregated descriptions rather than highly specific titles.

### Data collection

Twenty in-depth, semi-structured interviews were conducted in Georgian language, during fall 2024. Individual interviews lasted 45–60 min and were conducted either in person or via secure video conferencing. The Principal Investigator of this study and a trained public health researcher conducted all interviews. The interviewer was not involved in any of the participants' supervisory structures, minimizing the likelihood of bias. All interview were audio-recorded with consent, transcribed verbatim, and translated to English by the principal investigator, fluent in both languages.

### Data analysis

A deductive content analysis approach was used, structured around CFIR domains and constructs (22). An initial codebook was developed directly from CFIR. Transcripts were coded by the principal investigator, followed by grouping the coded data within each CFIR domain through iterative review. Specific quotes corresponding to these themes were extracted to provide illustrative examples of the identified gaps. To ensure anonymity in this small professional community, identifiers were dichotomized into “clinician” and “technical expert”.

### Ethics

Study approvals were obtained from the Ethics Committees of the National Center for Disease Control and Public Health of Georgia (NCDC&PH), the T. Tsertsvadze Infectious Diseases, AIDS, and Clinical Immunology Research Center (IDACIRC), as well as the National Center for Tuberculosis and Lung Diseases (NCTLD). Patients or members of the public were not involved in the design, conduct, reporting, or dissemination plans of this research. The study focused only on the perspectives of healthcare workers (HCWs) and relevant experts, directly engaged in TB preventive treatment and HIV care delivery in Georgia.

## Results

A total of 20 healthcare providers (17 female and 3 men) were interviewed:15 clinicians (13 representing HIV program, 2—TB program), one nurse, two program experts, and two policymakers from each program, respectively. The participants represented a total of 6 different facilities across the country: IDACIRC, three regional HIV facilities, NCTLD, and NCDC&PH. Based on participant responses and guided by the CFIR domains and their respective constructs, (Table 1) several potential barriers and facilitators to effective implementation of integrated electronic health systems were identified. The findings were organized and analyzed across the five CFIR domains: innovation characteristics, outer setting, inner setting, characteristics of individuals, and implementation process.

**Table 1:** Matrix summary of CFIR domains and constructs used for exploring the HCWs perspectives on potential implementation of an integrated electronic health records (EHRs) for TB and HIV programs.

CFIR Domains	Constructs
I. Innovation Characteristics	Source (innovation/program coming from a credible source)
	Evidence-base (has robust evidence supporting its effectiveness)
	Relative Advantage (is better than other available innovations or current practice)
	Complexity (is complicated, which may be reflected by its scope and/or the nature)
	Design (patient-centered service delivery, human resource, data system, the needed consumables, governance)
	Cost (operating costs are affordable)
II. Outer Setting	Policies and laws (legislation, regulations, professional)
	Local Conditions (economic, environmental, political, and/or technological)
	Financing (funding from external entities)
	Partnerships & Connections (facilities are networked with external entities)
	Critical Incidents (large-scale and/or unanticipated events disrupt implementation)
	External Pressure (Performance Measurement Pressure for quality or benchmarking metrics
III. Inner Setting	Structural characteristics (configuration of the inner environment and other tangible materials)
	Communications (formal and informal relationships, networks, information sharing practices)
	Culture (shared values, beliefs, and norms)
	Compatibility and relative priority (fitting within the current workflow, system, and process)
	Incentive system (tangible and intangible incentives and rewards and/or disincentives and punishments)
	Mission alignment (in line with the overarching goal of the HIV program)
	Resources (available resources to implement and deliver)
IV. Characteristics of Individuals	High-level leaders (decision-makers, executive leaders, directors)
	Mid-level leaders (leaders supervised by a high-level leader and who supervise others)
	Opinion leaders (individuals with informal influence on the attitudes and behaviors of others)
	Implementors (individuals with subject expertise, individuals leading implementation efforts, and other collaborators supporting EHR implementation and delivery)
	Deliverers and Recipients (individuals who directly or indirectly deliver or receive the services)
V. Process of Implementation	Teaming (the degree to which individuals team up, coordinate and collaborate on tasks)
	Needs assessment (priorities, preferences, and needs of people)
	Context assessment (collect information on current barriers and facilitators)
	Planning (identification of roles and responsibilities, outlining specific steps and milestones, defining goals and measures)
	Tailoring strategies (choosing and operationalization of implementation activities to address barriers, leverage facilitators and fit context)
	Engaging (attract and encourage participation of deliverers and recipients)
	Reflecting and Evaluating (collect and discuss quantitative and qualitative information about the success of implementation)

### Innovation characteristics

All HCWs recognized the relative advantage of an integrated EHR system over current practices, citing improved accessibility of patient information and the potential for more coordinated service delivery. Implementation of an integrated EHR was perceived as a solution to fragmented documentation and inefficient patient tracking, particularly for individuals on TPT. Respondents felt that a unified system would improve care continuity, reduce duplication of records, and support more informed decision-making.

However, a few HCWs raised concerns that enhancing interoperability between existing TB and HIV databases could inadvertently increase the risk of HIV-related stigma by exposing patients' HIV status more broadly across healthcare settings if appropriate safeguards were not ensured. These concerns reflected broader perceptions regarding confidentiality in shared digital environments rather than specific technical limitation of existing systems. This concern was particularly pronounced in HIV service provider facilities, opting for minimizing unnecessary disclosure. Interviewees noted that PWH often fear stigma and discrimination, increasing the likelihood of social harm, especially in smaller communities or facilities where personal information spreads easily without having proper safeguards.

While overall perspectives were largely consistent across TB and HIV program respondents, HIV-affiliated participants more frequently emphasized confidentiality and stigma-related concerns, whereas TB-affiliated participants tended to focus on operational and infrastructure-related challenges. These differences outlined contextual priorities rather than disagreement regarding the value of integration.

"The topic of a unified electronic system is definitely something to be organized—think this is very important those [the existing separate systems] to be integrated and thus patient follow-up efficiency improved." (R4, technical expert, male, 40–44 yrs)

"If this system brings everything together—TB and HIV—it will save time and allow for better tracking of important details and patient outcomes." (R16, clinician, female, 65–69 yrs)

"Many patients with HIV don't want their status known outside the HIV clinic. If we share systems with TB, it may cause them to stop coming altogether. Thus, ensuring confidentiality will be a key." (R9, clinician, female, 45–49 yrs)

"We have seen serious lapses where HIV status was disclosed to relatives without consent. This must not happen with an integrated database. Again, information sharing must be limited strictly to authorized individuals." (R8, clinician, female 50–54 yrs)

### Outer setting

An integrated EHR was seen to align with global donor priorities and national strategies advocating for service coordination between TB and HIV programs. Respondents highlighted support from international organizations such as the World Health Organization and the Global Fund as an important positive external factor that could accelerate EHR adoption. There was a perceived political will at the Ministry of Health level to support integration, although securing adequate financial resources was frequently mentioned as a prerequisite for successful and sustainable implementation. Nonetheless, many felt that these recommendations had yet to materialize into concrete policy directives or tangible support. Several respondents described the lack of national-level leadership and dedicated resources as key barriers to progress. Without their clear instructions, financial investment, or accountability mechanisms, efforts toward integration were viewed as aspirational but stalled.

"Probably, the external factors that may influence [EHR implementation] will be financial and legislative. Even if funding is allocated, issues will still arise due to existing confidentiality laws, especially given the sensitivity surrounding HIV status." (R2, clinician, female, 50–54 yrs)

"International organizations are heavily involved, sometimes even excessively, but their role is critical. They can support initiatives like EHR integration, although ultimately the government's leadership is essential." (R10, clinician, female, 40–44 yrs)

"I would highlight the positive role of international external factors. When international organizations are involved, attention is heightened and programs become far more effective, just like the experience with hepatitis C elimination program." (R7, clinician, male, 45–49 yrs)

"Financial factors will likely have the greatest impact. Steps like assembling the IT team and adapting the database won't succeed without proper funding." (R11, clinician, female, 50–54 yrs)

### Inner setting

Across the healthcare facilities interviewed, the HCWs expressed a generally positive organizational attitude toward the potential integration of TB and HIV electronic platforms. Many respondents indicated that at institutional level, there is generally strong willingness to adopt new innovations such as integrated EHR, driven by recognition of the benefits to patient management, program coordination, workflow efficiency, and overall performance metrics.

Nevertheless, substantial barriers were highlighted, particularly related to human resource constraints, physical infrastructure, and technical capacity, including functional computers, essential technological equipment, adequate server capacity, and data security systems. A major internal challenge identified was the shortage of trained personnel at different levels — not only clinicians but also IT specialists capable of system development, maintenance, data extraction, and continuous technical support. Respondents emphasized that implementing a robust EHR would require dedicated staff, financial investment in salaries, and extensive training across all facility levels. Without this, some feared the burden of managing a new system would fall disproportionately on already overextended healthcare workers.

A concern over increased workload during the transition phase was mentioned as another barrier. HCWs noted that adapting to a new system would initially be time-consuming and challenging, especially for regional facilities less experienced in digital transitions and database management, although they acknowledged that once integrated into daily practice, the system would ultimately simplify workflows by reducing redundant data entry and minimizing reliance on paper-based records. Some interviewees also cite limited internet connectivity, outdated hardware, and insufficient IT support as persistent barriers. In such settings, even basic data entry tasks were reported to be challenging, raising doubts about EHR sustainability.

Similarly to previous domains, confidentiality concerns were highlighted, underlying the importance of handling the sensitive HIV-related information. Despite these challenges, HCWs consistently expressed optimism about the long-term benefits of EHR integration, provided that sufficient financial, technical, and training support would be ensured.

"Our institution has always welcomed innovations. We continue to uphold that tradition; we see progress and are open to it." (R5, clinician, male, 35–39 yrs)

"There is a serious shortage of technical support. We would need dedicated staff for continuous maintenance and management. If you want something done properly, you need to pay for it, and for that, you need human, intellectual, and technical resources." (R1, clinician, female, 50–54 yrs)

"Implementation would require a big group of developers, since implementing all this will be very labor-intensive. Financial support and skilled personnel are crucial because we've seen poorly managed implementations fail." (R14, clinician, female, 45–49 yrs)

"The workload for doctors will significantly increase at first because they'll need time to learn all the details and adjust to the new system, but once it's part of the routine, it will significantly simplify their work." (R3, clinician, male, 30–34 yrs)

"This requires enormous financial and human resources. Besides, [EHR] creation and implementation is just the beginning—once implemented, the system will require continuous financial support and technical maintenance, and no matter how well-adapted the database is to program needs, it will always require some updates, changes, and reorganization." (R19, technical expert, female, 55–59 yrs)

### Characteristics of individuals

Healthcare workers consistently emphasized the importance of individual competence, motivation, and collaboration across all levels as essential to successful EHR implementation. A strong sense of professional readiness and technical capacity was reported on the implementors' level, with many respondents expressing confidence in their experience with data systems and general openness to innovations. Respondents commonly believed that, given clear guidance from higher-level authorities and adequate training, they would be able to adapt to the new system effectively.

Participants also underscored the importance of strategic leadership and stakeholder coordination. Several respondents stated that involvement from upper-level decision-makers, especially at the Ministry of Health and other regulatory institutions, would be essential to drive implementation and ensure financial and institutional support. The role of mid-level management (e.g., supervisors, department heads) was also seen as vital by all participants in both—the design and operational phases of the system. Their involvement in early planning was viewed as key to identifying workflow needs and facilitating downstream adoption.

Despite broad professional support, a concern over digital literacy gap among older clinicians was identified as one of the barriers. Previous negative experiences with underdeveloped or poorly supported data systems were noted, highlighting the importance of clinician involvement, ongoing technical assistance, and post-rollout support.

"We all know the current programs inside out. Transferring them to an electronic format shouldn't be difficult and doctors won't oppose the idea. It will simplify coordination between TB and HIV programs." (R2, clinician, female, 50–54 yrs)

"If we clearly present the problem and articulate the benefits of solving it, I'm sure upper-level decision-makers will be receptive. It all depends on how we communicate our needs." (R1, clinician, female, 50–54 yrs)

"The Ministry's role as a decision-maker is the most important—if the initiative and directive come from them, everything else will follow. Local supervisors will ensure compliance, and doctors will be trained respectively." (R10, clinician, female, 40–44 yrs)

"We've had cases where poor communication between developers and medical staff led to glitches that slowed us down. The smallest overlooked detail can disrupt daily work." (R14, clinician, female, 45–49 yrs)

"The workforce is somewhat aging, and many lack digital skills. It's not hopeless, but they'll need regular support following the initial training." (R19, technical expert, female, 55–59 yrs)

### Process of implementation

Healthcare workers described a range of important considerations regarding the process of implementing an integrated EHR system for TB and HIV services in Georgia. Overall, participants recognized that careful planning, engagement, and coordination would be necessary for successful implementation.

The creation of a multidisciplinary working group was recurringly recognized as a facilitator, ideally composed of clinicians, data managers, IT developers, policymakers, and technical experts representing each program. This team would be responsible for co-designing the system, defining specific requirements for the integrated EHR, and aligning their objectives of improving patient outcomes. Several HCWs emphasized that coordinated, cross-functional planning and stakeholder input—especially from those managing data on the ground—was crucial for developing a system that reflects actual clinical and operational needs.

Another commonly identified requirement was a phased implementation strategy, beginning with infrastructure and needs assessment, followed by piloting, workforce training, and iterative feedback mechanisms. HCWs also stressed the importance of dedicated liaison personnel, who could filter user issues, relay them to developers, and provide structured responses to clinicians. This was considered essential for minimizing miscommunication, maintaining user engagement, and reducing implementation fatigue.

However, without adequate cultural readiness, technological equipment, appropriate planning, and adequate financial resources, including staff incentivization, respondents feared that a rushed deployment could undermine long-term sustainability. Many also noted the need for tailored training programs for clinicians and nurses, emphasizing that even those with prior data entry experience would need system-specific guidance to ensure uniform data quality and adoption. Again, concerns over confidentiality and the sensitive nature of HIV-related data were noted as essential issues to be addressed during the planning phase. Participants emphasized that EHR systems must be designed with robust privacy safeguards and user permissions to protect patient identity and reduce stigma-related fears.

"We must assess our infrastructure and needs. Based on the level of integration required, we can select the most suitable model, followed by technical specification, training, piloting, implementation, and back to workflow reassessment. " (R19, technical expert, female, 55–59 yrs)

"Training is essential since every new system has its own specificities. Even if we already work with data, we'll still need to be retrained for this. " (R18, clinician, female, 60–64 yrs)

"First, we need a working group with both programmers and healthcare professionals. If we don't have a well-argued, structured plan to show upper management, no one will even bother thinking about it." (R11, clinician, female, 50–54 yrs)

"There should be an intermediary contact person who filters issues between users and developers. It's ineffective to connect every doctor directly with the developer team." (R6, technical expert, female, 50–54 yrs)

"A poorly designed system without proper programming support can be worse than doing nothing at all. It should make life easier for doctors—not harder." (R9, clinician, female, 45–49 yrs)

"Planning should begin by ensuring patient confidentiality. Even in registration/referral forms, term like ‘AIDS center' should be avoided to protect the patient." (R15, clinician, female, 50–54 yrs)

## Discussion

This qualitative study provides critical insights into perceived barriers and facilitators to implementing an integrated electronic health record system within TB and HIV programs in the country of Georgia. The findings reveal that the potential benefits of an integrated EHR greatly outweigh possible barriers, such as legal restrictions, resource shortages, and conceivable resistance to change. While integration of TB and HIV services has been examined in several settings, there is limited data exploring the digital and organizational dimensions of such integration in the Eastern Europe and Central Asia (EECA) region. To our knowledge this is among the first papers to systematically apply an implementation science framework to HIV-TB service integration in this context. Although context-specific, the findings have broader implications for digital health system implementation across diverse disease areas and care settings globally.

Our findings align global evidence highlighting the potential of integrated electronic health information systems, such as integrated EHR, for enhancing patient tracking, streamlining data collection, and improving coordinated care delivery (24, 25). Participants identified several anticipated benefits of integrated EHR, particularly in improving data completeness, reducing fragmented documentation, and enhancing continuity of care, reflecting broader international experiences (25–29). These observations closely match experiences reported in earlier studies that found integrated EHR systems to substantially improve care coordination, clinical decision-making, and overall patient management despite various infrastructural challenges (30, 31). This highlights that fragmented systems hinder patient follow-up and distort performance metrics, masking real progress in TPT uptake and limiting accurate program evaluation. Integration of HIV and TB platforms with real-time exchange are therefore essential for both service delivery and reliable monitoring. Concerns surrounding patient confidentiality, privacy, and stigma emerged as critical barriers among our study respondents. Notably, this study did not assess the specific technological safeguards of the national EHR, rather the concerns reflect stakeholder perceptions regarding potential risks of unauthorized disclosure in shared digital environments. The perceptions raised highlight the need for robust safeguards, including role-based access controls, clear data governance policies, and secure information-sharing protocols in any future interoperable systems. This mirrors findings from 2022 study by Alzghaibi and Hutchings, who highlighted similar privacy and confidentiality issues severely impacting EHR adoption, also comparable with a review by Farzianpour et al., where confidentiality concerns significantly limited user acceptance and further utilization of electronic health platforms (32, 33). Different, but significant implementation barriers were also identified across literature, including limited interoperability, insufficient training, and infrastructural inadequacies, which also resonate with challenges faced in Georgia (24, 33, 34).

Key contextual factors identified among our respondents included support from international organizations, importance of clear national policies and structured governance, adequate funding, and additional human resources to support successful EHR implementation. These findings are consistent with evidence from published literature, including from the European context, where fragmented governance, insufficient financial resources, and limited stakeholder engagement significantly impeded the implementation of an integrated electronic platform (28, 35). Comprehensive and iterative workforce training emerged as crucial for successful EHR implementation, consistent with other reported evidence (26). In our study, these concerns referred to the potential implementation of an interoperable TB/HIV electronic system and were largely informed by participants' prior experiences with introducing new reporting systems within their programs. Respondents also emphasized phased implementation approaches, including infrastructure assessments, piloting, and iterative feedback mechanisms, aligning with global best practices, although highlighting implementation barriers related to significant human resource constraints, including clinical and technical personnel, as well as existing digital literacy gap concerning the capabilities of system development, data systemization, and continuous technical support required for EHR maintenance.

A key strength of our study is the rigorous, CFIR-guided qualitative methodology, which enabled structured comprehensive exploration of individual and system-level implementation factors (23, 36). However, potential qualitative biases and geographical specificity may affect the generalizability of findings. However, these findings may be transferable to other EECA settings with similar siloed TB/HIV structures and constrained digital infrastructures. As researchers with professional familiarity with Georgia's TB and HIV programs, we remained attentive to our positionality and its potential influence on interpretation. The results reflect CFIR-predicted multilevel determinants, while also highlighting context-specific nuances that extend existing implementation evidence, particularly, around confidentiality and resource constraints.

In summary, our study highlights several policy and programmatic priorities with relevance beyond TB and HIV services. First, implementing robust confidentiality protections and stigma-reduction efforts is essential to foster trust in electronic systems and support user uptake. The potential development of an EHR must be grounded in strong data protection policies that safeguard anonymity, ensure secure handling of sensitive information, and clearly delineate access to confidential data. Second, clearly defined national policy frameworks and sustained financial support are critical to ensure long-term EHR functionality and scale-up. Thus, it's crucial to inform policymakers, program implementers, and other decision-makers to facilitate and consistently support EHR implementation. Third, comprehensive and context-specific training programs should be implemented to strengthen digital competencies among diverse healthcare workers and promote system engagement. Finally, phased implementation strategies, including infrastructure assessments, pilot testing, and continuous feedback can help anticipate context-specific challenges and support adaptive, sustainable system integration.

This study has several limitations. The sample included all credible and available stakeholders engaged in TPT and HIV program activities in Georgia, but perspectives from individuals outside these structures may be underrepresented. Social desirability bias and self-censorship remain possible within a small, interconnected professional community, and the absence of patient perspectives, particularly regarding confidentiality concerns, represents an important gap for future exploration. As with most qualitative work, findings are context-specific and should be applied cautiously to settings with different organizational or digital infrastructures.

In conclusion, the insights gained from this study can guide the selection of effective implementation strategies to overcome identified barriers. These barriers include concerns about patient confidentiality, securing adequate financial resources, establishing clear governance structures, and providing comprehensive training. Additionally, the study emphasizes the importance of phased implementation and the use of iterative methods for continuous improvement of processes and products. This approach will facilitate the successful implementation of integrated EHR systems in Georgia.

## Data Availability

The original contributions presented in the study are included in the article/Supplementary Material, further inquiries can be directed to the corresponding author.
