# Supplementary material for: Barriers and facilitators to implementing an integrated electronic health records system to improve tuberculosis preventive treatment among people living with HIV: a content analysis study from Georgia
Source: Front Digit Health. 2026 May 4;8:1754076. doi: 10.3389/fdgth.2026.1754076 (PMC13180559; doi:10.3389/fdgth.2026.1754076)
Supplement: Supplementary file 1 [file Table1.docx]

**A guide for in-depth interview among TB/HIV service providers in Georgia**

*The interviewer explains the overall study objectives and procedures for participation, including providing information about the audio recording, and obtains written informed consent from the participant.*

**Introduction**

Thank you for agreeing to participate in this in-depth interview. You have been briefed about the study goal, alongside the guidelines and rights that come with your involvement in this research. Your written consent to participate has also been collected. Now, we are ready to begin.

You are informed that the study's focus is on assessing the context for implementing tuberculosis preventive treatment in HIV clinics, including TPT characteristics and related contextual factors at individual (provider), clinic (inner setting), and outer setting/system levels, and additionally, assessing the context for a potential implementation of an evidence-based practice for improving data recording and reporting on TPT in HIV clinics. As recognized in the preliminary results of our previous study looking at TB incidence rate among PLHIV, some major gaps were identified, specifically, within the recording and reporting of the majority of the clinical data, including baseline TB screening following the HIV diagnosis, TPT eligibility assessment, and TB preventive treatment initiation. Within previous interviews on barriers and implementations for TPT scale-up, an absence of integrated electronic health records (EHR) system within T/HIV programs was highlighted as a significant factor, that could have potentially improved the overall provision of the program, including enhanced patient adherence monitoring and follow-up. Thus, the insights from these interviews will significantly contribute to the knowledge of TB preventive strategies, offering insights on existing barriers and facilitators to the operationalization of the TPT program in a real-world setting and the potential of EHR implementation. The experiences and recommendations gained from the healthcare workers will aid in informing policymakers, program implementers, and help shaping future services and optimization of TB prevention and care, as well as data recording and reporting among PLHIV.

I would like to remind you that our interview is expected to last for about 40-60 minutes. Some questions might seem sensitive or very personal to you. Please remember that you have the liberty to skip any question that you might find uncomfortable or to stop your participation at any moment. If you have any questions or need further explanation during the interview, feel free to ask. This interview will be audio-recorded for transcription and analysis. The recording will strictly be for our internal use, and your confidentiality will be fully protected. Please, if you have any questions or concerns about the process, share them with me now. Also, kindly reaffirm your consent to participate before we proceed.

| **Exploring the barriers and facilitators to implementing an integrated TB/HIV electronic data system in HIV care settings** |
| --- |
| **Introduction:** *[the interviewer will explain that this part of the interview specifically focuses on exploring the barriers and facilitators to implementation of an integrated TB/HIV electronic data system (including electronic medical records, EMR) for TPT-related data and define that implementation of EMR has been shown to be effective in several studies – including a reference to the 1^st^ quantitative manuscript, with results showing major gaps and limitations in this area, and describing relevant details]* |
| **Domain – Innovation characteristics** *(evaluating understanding of the potential intervention [EMR system] as perceived in terms of its features, complexity, and adaptability)* |
| 1. What do you think about implementing an integrated electronic medical records system (EMR) in general, for medical information recording and reporting purposes within the HIV program, including TPT? Probe on the following:    - *Source (organization, that developed and/or visibly sponsored use of innovation, is reputable, credible, and/or trustable)*    - *Evidence-based (has robust evidence supporting its effectiveness)*    - *Relative Advantage (is better than other available innovations or current practice)*    - *Innovation adaptability and trialability (innovation can be tested or piloted on a small scale and undone/modified, tailored, or refined to fit local context or needs)*    - *Complexity (is complicated, which may be reflected by its scope and/or the nature and number of connections and steps)*    - *Design (is well designed considering: patient-centered service delivery, human resource needs/ capacity, data system needs/capacity, the needed drugs/tests/consumables, governance)*    - *Cost (operating costs are affordable)* |
| **Domain – Outer setting** *(explore external factors that could influence the implementation of EMR, such as economic, political, and technological factors)* |
| 1. What do you think about external conditions to as barriers/facilitators to implementation of integrated EMR within the HIV program? Probe on the following:    - *Policies and laws (legislation, regulations, professional group guidelines and recommendations, or accreditation standards support implementation and/or delivery of the innovation)*    - *Local Conditions (economic, environmental, political, and/or technological conditions enable the MOH/ NCDC/ IDACIRC/ NCTLD to support implementation of integrated EMR within the HIV program)*    - *Financing (funding from external entities [e.g., GF] is available to implement and/or deliver the innovation)*    - *Partnerships & Connections (HIV clinics/ facilities are networked with external entities, including referral networks, academic affiliations, and professional organization networks)*    - *Critical Incidents (large-scale and/or unanticipated events, e.g., COVID19, disrupting innovation implementation)*    - *External Pressure (e.g., Performance Measurement Pressure for quality or benchmarking metrics or established service goals drive implementation and/or delivery of the innovation, e.g., from GF, WHO, MOH)* |
| **Domain – Inner setting** *(examine the internal environment, including organizational culture, readiness for integrated EMR implementation, and resources)* |
| 1. What do you think about the internal environment for potential implementation of TB/HIV integrated electronic platform within the HIV program? Specifically:    - *Structural characteristics (configuration of the inner environment and other tangible materials, including technological systems for communication, documentation, and data storage, management, reporting, organizational tasks and responsibilities within and between individuals and teams, that support functional performance of the EMR)*    - *Communications (formal and informal relationships, networks, information sharing practices within and across the inner environment related to EMR implementation and enhancement)*    - *Culture (shared values, beliefs, and norms around caring, supporting, and addressing the needs of EMR program users, i.e., HCWs, administrative personnel, researchers, and around psychological safety, continual improvement, and using data to inform practice)*    - *Tension for change (the degree to which the current situation is intolerable and needs to change)*    - *Compatibility and relative priority (does EMR fit within the current workflow, system, and process and is its’ delivery more important compared to other initiatives?)*    - *Incentive system (are there tangible and intangible incentives and rewards and/or disincentives and punishments supporting implementation of EMR?)*    - *Mission alignment (implementation of EMR is in line with the overarching goal of the HIV program)*    - *Resources (available resources to implement EMR: funding, human resources, supplies, guidance and/or information and knowledge access, e.g., training, etc.)* |
| **Domain – Characteristics of individuals** *(assess the roles, attitudes, and skills of the individuals involved in the implementation process)* |
| 1. Discuss the roles, competence, knowledge, skills, availability, and commitment of individuals with different levels of authority regarding EMR implementation:    - *High-level leaders (decision-makers, executive leaders, directors)*    - *Mid-level leaders (leaders supervised by a high-level leader and who supervise others)*    - *Opinion leaders (individuals with informal influence on the attitudes and behaviors of others)*    - *Implementors (individuals with subject expertise, individuals leading implementation efforts, and other collaborators who support the EMR implementation and delivery in practice)*    - *Deliverers and Recipients (individuals who directly or indirectly deliver or receive EMR services)* |
| **Domain – Process of implementation** *(focus on the steps taken during the implementation process, including planning, engaging, and evaluating)* |
| 1. Describe the activities and strategies necessary to implement EMR at your clinic? Probe on the following:  - *Teaming (the degree to which individuals team up, coordinate and collaborate on tasks to support EMR implementation)* - *Needs assessment (priorities, preferences, and needs of people, i.e., HCWs, administrative personnel, researchers, etc.)* - *Context assessment (collect information on barriers and facilitators to EMR implementation)* - *Planning (identification of roles and responsibilities, outlining specific steps and milestones, defining goals and measures for EMR implementation success)* - *Tailoring strategies (choosing and operationalization of implementation activities to address barriers, leverage facilitators and fit context)* - *Engaging (attract and encourage participation of EMR deliverers and recipients)* - *Doing (EMR implementation in small steps, tests, or cycles of change, to trial and cumulatively optimize implementation strategies)* - *Reflecting and Evaluating (collect and discuss quantitative and qualitative information about the success of EMR and its’ implementation)* - *Adapting (modify EMR implementation strategies and/or inner environment for optimal fit and integration into work process)* |
| **Summary questions** *(This last section of our interview guide will close the interview and allow the participants to express their final thoughts about the topic)* |
| 1. What changes would you recommend supporting the implementation of integrated TB/HIV data system in Georgia? 2. Would you like to add/recommend anything related to TB/HIV data system including EMR, that we have not covered during the interview? |

Now, we are concluding our interview. Thank you for your valuable participation. If you have anything else to add that we have not covered, please feel free to share. If you have any further questions or concerns, please contact me any time via email or phone.
